# CONFLICT MONITORING IN AMNESTIC MILD COGNITIVE IMPAIRMENT: FINDINGS FROM A PICTURE–WORD INTERFERENCE PARADIGM

**DOI:** 10.1093/geroni/igae098.3310

**Published:** 2024-12-31

**Authors:** Elizabeth Lydon, Raksha Mudar

**Affiliations:** University of Illinois Urbana-Champaign, Champaign, Illinois, United States; University of Illinois, Champaign, Illinois, United States

## Abstract

Older adults with amnestic mild cognitive impairment (MCI) experience a decline in episodic memory but do not meet diagnostic criteria for dementia. Mounting evidence suggests other aspects of cognition may be impacted, including behavioral and neural alterations in conflict monitoring. Neural underpinnings of these changes have been captured through the use of electroencephalography (EEG) by most frequently examining event-related potentials (ERPs). Fewer studies have examined event-related spectral perturbations (ERSPs) that underlie conflict processes in MCI. We examined differences between 27 individuals diagnosed with amnestic MCI (age: 75.3 ± 5.6) and 27 age- and education-matched cognitively healthy controls (HC; age: 72.6 ± 7.9) using a novel picture-word interference paradigm. Stimuli for this task included line drawings of animals and objects with matched or mismatched word labels. Participants judged if the picture-word pairs were matched or mismatched using a response box while continuous EEG was recorded. We examined differences between HC and MCI on behavioral performance (accuracy and reaction time) and ERSP measures (mean spectral power in theta, alpha, and beta frequency bands). Behavioral results reveled that the MCI group took longer to respond and was less accurate than the HC group on mismatched (conflict) trials across both tasks. Additionally, both groups were less accurate when responding to mismatched versus matched trials across tasks. Preliminary analysis of ERSP measures showed different patterns of neural oscillatory activity within and across the two groups. Findings support the presence of behavioral and neural alterations in conflict processing in amnestic MCI.

